# Flexible Freestanding Carbon Nanofiber-Embedded TiO_2_ Nanoparticles as Anode Material for Sodium-Ion Batteries

**DOI:** 10.1155/2018/4725328

**Published:** 2018-11-04

**Authors:** Xuzi Zhang, Zhihong Chen, Lingling Shui, Chaoqun Shang, Hua Liao, Ming Li, Xin Wang, Guofu Zhou

**Affiliations:** ^1^National Center for International Research on Green Optoelectronics, South China Normal University, Guangzhou, China; ^2^Shenyang Institute of Automation, Chinese Academy of Sciences, Guangzhou, China; ^3^Institute of Solar Energy, Yunnan Normal University, Kunming, China; ^4^International Academy of Optoelectronics at Zhaoqing, South China Normal University, Guangdong, China

## Abstract

Sodium-ion batteries (SIBs), owning to the low cost, abundant resources, and similar physicochemical properties with lithium-ion batteries (LIBs), have earned much attention for large-scale energy storage systems. In this article, we successfully synthesize flexible freestanding carbon nanofiber-embedded TiO_2_ nanoparticles (CNF-TiO_2_) and then apply it directly as anode in SIBs without binder or current collector. Taking the advantage of flexible CNF and high structural stability, this anode exhibits high reversible capacity of 614 mAh·g^−1^ (0.27 mAh·cm^−2^) after almost 400 cycles and excellent capacity retention ability of ~100%

## 1. Introduction

Sodium-ion batteries (SIBs) have earned much attention as a candidate substitution for lithium-ion batteries (LIBs) in the area of large-scale energy storage [1, 2], which is ascribed to the earth's abundance of sodium resource and similar physicochemical properties with LIBs [3–5]. Until now, many efforts have been made to solve the slow sodiation/desodiation kinetics and large-volume expansion caused by a large radius of Na^+^ (1.02 Å versus 0.76 Å of Li^+^) [6–8]. Furthermore, the capacity and cycling stability also need to be improved to satisfy the practical application. It is essential to select and design proper anode materials for SIBs to realize fast Na^+^ insertion/extraction with high capacity and cycling stability [9, 10].

Among numerous anode material candidates, TiO_2_ with anatase phase has been explored as a promising anode material for SIBs with low cost, abundance, environmental benignity, and excellent structural stability [11–13]. However, the undesirable electrical conductivity and sluggish ionic diffusivity restrict its further applications [14]. Many efforts have been cost to improve the ion/electron transportation for SIBs. Zhu and coworkers [15] synthesized TiO_2_ nanoparticles coated by mutiwalled carbon nanotubes and carbon nanorods as anode, exhibiting excellent rate capability and cycling stability. He and coworkers [16] prepared a hierarchical rod-in-tube structure TiO_2_ modified with a conducive carbon layer as anode, which delivered fast ion diffusion and high conductivity. Therefore, the efficient strategy to enhance the electrochemical performance is nanosizing TiO_2_ and then incorporating with the conductive matrix [16–22]. Despite the progresses, the rational design nanostructure of TiO_2_-based anode is still of great demand.

Herein, we proposed freestanding flexible winkled carbon nanofiber-embedded anatase TiO_2_ nanoparticles (CNF-TiO_2_) as anode of SIBs directly without binder and current collector, which can not only increase the energy density but also explore the potential application in flexible devices. The long-range continuous carbon nanofibers can improve the conductivity of nanosized anatase TiO_2_, and the thin fibers can shorten the diffusion path of Na^+^, which can promote the electrochemical kinetics in Na^+^ insertion/extraction. The freestanding flexible 3D carbon structure and embedded TiO_2_ nanoparticles can improve structural stability to alleviate the volume change during Na^+^ insertion/extraction. In addition, the rough surface of CNFs increases the electrode-electrolyte contact points and lowers the charge transfer resistance. High specific capacity of 614 mAh·g^−1^ (0.27 mAh·cm^−2^) was obtained after almost 400 cycles with capacity retention of ~100%, confirming the potential of CNF-TiO_2_ as anode for SIBs.

## 2. Experimental Section

### 2.1. Synthesis of the Freestanding CNF-TiO_2_

The electrospinning precursor solution was prepared firstly by dissolving 1.48 g polyacrylonitrile (PAN, Mw = 150,000, Sigma-Aldrich) in 18 ml *N*,*N*-dimethylformamide (DMF) under magnetic stirring overnight. Then, 2.5 ml tetrabutyl titanate (Ti(OC_4_H_9_)_4_) was added into this solution and stirring was continued for 10 min to obtain homogeneous white turbid solution. The distance between the needle and Al foil was 15 cm, and the voltage was maintained at 25 kV. Then the obtained precursor nanofibers were stabilized at 280°C for 2 h with a heating rate of 5°C·min^−1^ and carbonized at 700°C with 1°C·min^−1^ for 2 h under argon atmosphere.

### 2.2. Structure Characterizations

The morphologies and size of CNF/TiO_2_ were characterized by scanning electron microscopy (SEM, ZEISS Ultra 55). Transmission electron microscopy (TEM) and EDS mapping were both carried out by JEM-2100 HR. The crystalline property of CNF-TiO_2_ was recorded by Bruker D8 Advance. The thermal gravity analysis TG test was performed to evaluate the content of TiO_2_ by Netzsch STA 449. The 250Xi X-ray photoelectron spectroscope (XPS) was obtained from ESCALAB.

### 2.3. Electrochemical Tests

The CR2016-type coin cells were assembled with sodium metal as the reference electrode, glass fiber membrane as the separator, and the as-prepared CNF-TiO_2_ directly as the anode. The above procedures were all carried out in an Ar-filled glove box (O_2_ < 0.1 ppm, H_2_O < 0.1 ppm). The electrolyte was 1 M NaClO_4_ in propylene carbonate (PC)/ethylene carbonate (EC) (PC : EC = 1 : 1, in volume). The cyclic voltammetry (CV) and electrochemical impedance spectroscopy (EIS) results were obtained from an electrochemical workstation (CHI660E, Shanghai Chen Hua Instruments Ltd). Also, the galvanostatic discharge-charge tests were conducted in a Neware battery testing system.

## 3. Results and Discussion

**Figure 1:**
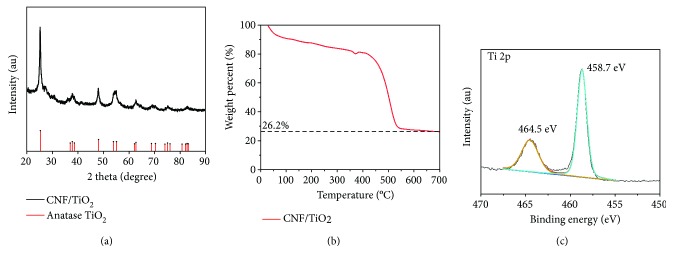
(a) The XRD pattern of CNF-TiO_2_ after pyrolysis at 700°C; (b) TG pattern of CNF-TiO_2_ under air atmosphere; (c) XPS of Ti 2p in CNF-TiO_2_.

The structure and morphology of CNF-TiO_2_ are detected by XRD, TG, XPS, SEM, and TEM. As shown in Figure 1(a), all the diffraction peaks matched well with anatase TiO_2_ (JCPDS number 021-1272), which confirms that the pyrolysis temperature is appropriate to gain high-purity anatase TiO_2_. Furthermore, the slightly weak intensity of these diffraction peaks suggests that the TiO_2_ nanoparticles were well embedded in the carbon nanofibers. In the thermogravimetry measurement (Figure 1(b)) of CNF-TiO_2_, the content of TiO_2_ is 26.2%. The Ti in CNF-TiO_2_ is clarified by X-ray photoelectron spectroscopy (XPS) as shown in Figure 1(c), which indicates two peaks of 464.6 eV and 458.7 eV, corresponding to the orbits of 2p 3/2 and 2p 1/2 of Ti^4+^, respectively. The Ti 2p XPS result also confirms the formation of anatase TiO_2_.

**Figure 2:**
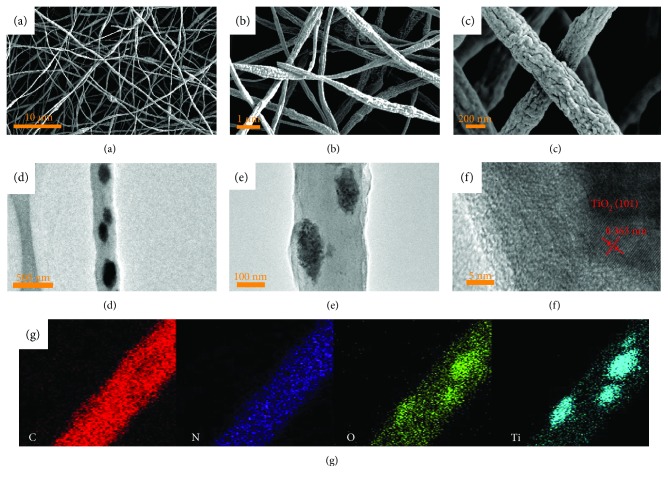
SEM images (a–c), TEM images (d–f), and EDS mapping (g) of CNF-TiO_2_.

Figures 2(a)–2(f) perform the morphologies of CNF-TiO_2_. SEM images (Figures 2(a)–2(c)) show an extremely rough surface of the as-synthesized nanofibers with diameter of ~300 nm. Many wrinkles appear after 700°C pyrolysis treatment for the crystallization of TiO_2_ nanoparticles and decomposition of the polymer fibers, which may provide active sites for Na^+^ insertion/extraction. In addition, the long-range continuous carbon nanofiber matrix with high conductivity will lead to fast electron transmission. As for the TEM images with different magnification (Figures 2(d)–2(f)), the well-distributed TiO_2_ nanoparticles can be clearly observed with sizes between 100 nm and 200 nm and they are all coated with amorphous carbon. A lattice spacing of 0.363 nm, corresponding to (101) planes of anatase TiO_2_, can be clearly detected in the high-resolution TEM image (Figure 2(f)), which means the high degree of crystallinity of anatase TiO_2_. The TiO_2_ larger lattice spacing of 0.363 nm than 0.102 nm of Na^+^ and the specific space group of I4_1_/*amd* (*a* = 3.785 Å, and *c* = 9.514 Å) can ensure the fast insertion/extraction of Na^+^ [23, 24], in which Na^+^ is inserted/extracted in the interspace of anatase TiO_2_ [23]. Furthermore, it can stabilize the structure of CNF-TiO_2_ cooperated with amorphous carbon through enduring the volume change in the battery reaction.

**Figure 3:**
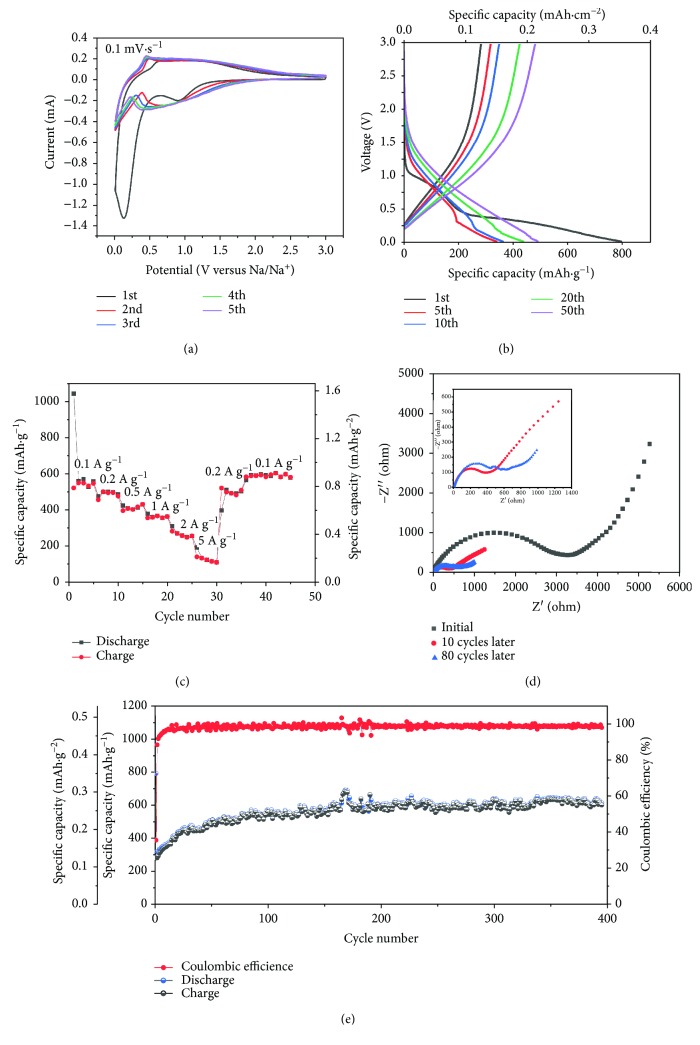
(a) CV tests at 0.1 mV·s^−1^. (b) Galvanostatic charge-discharge curves of CNF-TiO_2_ recorded at 200 mA·g^−1^; (c) rate performance of CNF-TiO_2_; (d) EIS of CNF-TiO_2_ before and after cycles; (e) cycling stability of CNF-TiO_2_ as anode for SIBs at 200 mA·g^−1^.

The electrochemical performance of CNF-TiO_2_ anode for SIBs is investigated by cyclic voltammetry (CV) between 0.01 V and 3 V with a scan rate of 0.1 mV·s^−1^ and galvanostatic charge-discharge techniques at 200 mA·g^−1^. As depicted in Figure 3(a), a strong cathodic peak between 0 and 0.5 V appears in the first cycle of SIB and disappears in the following four cycles, which demonstrates the decomposition of electrolyte and the formation of solid electrolyte interphase (SEI) film. The benign overlapped CV curves of the next four scans indicate excellent cycle stability and reversibility for Na^+^ insertion/extraction.  Figure 3(b) shows the charge/discharge curves of CNF-TiO_2_ as anode for SIBs with constant current of 200 mA·g^−1^. In the initial cycle, there exists a large irreversible capacity compared to the following curves, which is in agreement with the CV tests. The charge/discharge curves without obvious plateaus demonstrate the fluent insertion/extraction of Na^+^ into the amorphous carbon and crystalline TiO_2_ lattice. The initial discharge capacity is 792 mAh·g^−1^ (0.35 mAh·cm^−2^) with a coulombic efficiency of 35.5%, and the discharge capacities increase slightly during the subsequent cycles, showing the continuous reduced resistance of CNF-TiO_2_ by the activation of this material, which is also confirmed in electrochemical impedance spectroscopy (EIS, Figure 3(d)). The EIS results show the slight decrease in charge transfer resistance before 10 cycles and then a gradual increase until 80 cycles, which is the consequence of activation and slight structural damage of CNF-TiO_2_, respectively.

The rate performance of CNF-TiO_2_ is further investigated at various constant currents from 100 mA·g^−1^ to 5000 mA·g^−1^. As shown in Figure 3(c), the capacity can still retain 378 mAh·g^−1^, 309 mAh·g^−1^, and 133 mAh·g^−1^ at the current densities of 1000 mA·g^−1^, 2000 mA·g^−1^, and 5000 mA·g^−1^, indicating the rapid process of the insertion/extraction of Na^+^. Moreover, when the current density recovers to 100 mA·g^−1^, the capacities can retain to the initial level, showing the outstanding rate performance of CNF-TiO_2_ as anode for SIB. CNF-TiO_2_ also exhibits remarkable long-term cycling stability (Figure 3(e)). It can deliver a high initial capacity of 792 mAh·g^−1^ with a coulombic efficiency of 35.5% and stability at 614 mAh·g^−1^ after almost 400 cycles, indicating the excellent cycling performance and structural stability of CNF-TiO_2_ anode. On the one hand, the large length-to-volume ratio of CNFs-TiO_2_ provides more active sites for Na ion adsorption on the surface of 1D nanofibers, which offers additional capacity contribution. On the other hand, the specific capacity of CNFs-TiO_2_ is based on the mass of TiO_2_, while the carbon substrate may contribute partial capacity. It should be noted that the capacity increases during the initial cycles, which might be attributed to the active process owing to the 3D interconnected nanostructure of CNF-TiO_2_ [16].

**Figure 4:**
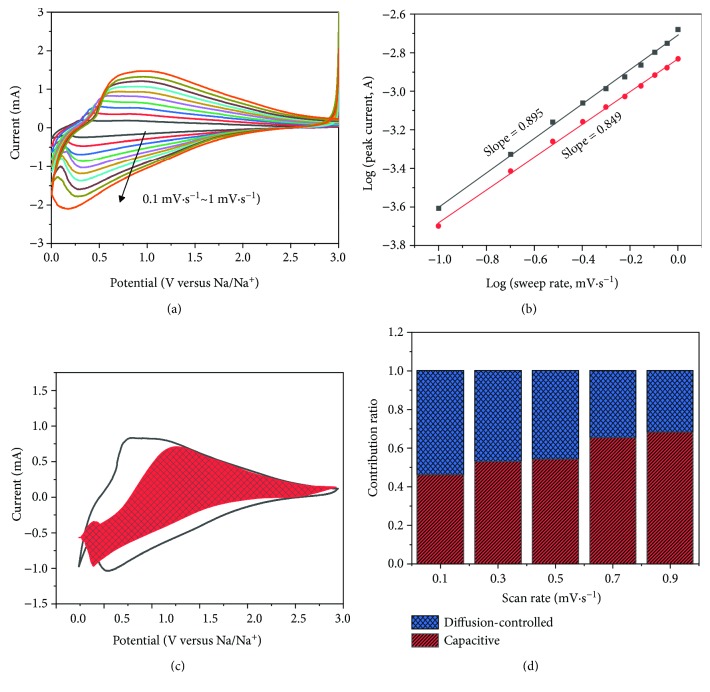
(a) CV curves of CNF-TiO_2_ at different scan rates from 0.1 mV·s^−1^ to 1 mV·s^−1^. (b) The relationship between peak current (i) and scan rates (v). (c) The contribution of capacitive (red) and battery (blank) reaction at 0.5 mV·s^−1^. (d) The ratio of capacitive and battery contribution at different scan rates.

To further unravel the electrochemical kinetic properties of CNF-TiO_2_ as anode in SIBs, CV tests at different scan rates from 0.1 mV·s^−1^ to 1 mV·s^−1^ are performed in Figure 4(a). All the CV cycles have a similar shape of broad peaks for Na^+^ insertion/extraction. Also, the small peak shift with different scan rates indicates the smaller polarization of CNF-TiO_2_. The peak current (*i*) of curves can be separated into two mechanism parts: diffusion-controlled and surface-controlled, which corresponds to battery and capacitive reaction, respectively.

In order to figure out the contribution of each part, the equation of *i* = *a* · *v*^*b*^ [25, 26] linked peak current (*i*, mA) and scan rate (*v*, mV·s^−1^) is performed to qualitatively analyze the kinetics, which can also express as log *i* = log *a* + *b*·log *v*. *a* and *b* are constants which are obtained from the experiments. The *b* value is represented by the slope of log *v* − log *i* plots. There are two limit cases: that *b* = 0.5 means a diffusion-controlled mechanism (battery) and that *b* = 1 represents a surface-controlled process (capacitive). As shown in Figure 4(b), the cathodic peaks show the estimated *b* value of 0.895 and anodic peaks of 0.849 from 0.1 mV·s^−1^ to 1 mV·s^−1^, which means the electrochemical kinetic of CNF-TiO_2_ as anode is the combined mechanism of diffusion control and surface control (dominant).

Furthermore, the capacitive contribution and battery contribution can be separately quantitatively analyzed by the equation *i* = *k*_1_*v* + *k*_2_*v*^1/2^ [20], where *i* is the current at a fixed voltage with different scan rates, and *k*_1_*v* and *k*_2_*v*^1/2^ originated from the contribution of surface-controlled and diffusion-controlled reaction, respectively. In order to easily calculate, this formula can be transformed to *i*/*v*^1/2^ = *k*_1_ *v*^1/2^ + *k*_2_. Then, *k*_1_ and *k*_2_ can be obtained from the fitting plot of *v*^1/2^ − *i*/*v*^1/2^.  Figure 4(c) shows that the current is derived from two parts with the obvious red shadow area and blank space representing capacitive and battery reaction, respectively, which indicates that the contribution of capacitive effect is 54.3%.  Figure 4(d) exhibits the capacity contribution increasing with the rising scan rates, 46.2% (0.1 mV·s^−1^), 53% (0.3 mV·s^−1^), 54.3% (0.5 mV·s^−1^), 65.4% (0.7 mV·s^−1^), and 68.4% (0.9 mV·s^−1^). These capacitive contributions reveal that CNF-TiO_2_ as anode can shorten the electron transfer path and decrease the barrier of Na^+^ insertion/extraction.

## 4. Conclusion

In summary, this flexible freestanding CNF-TiO_2_ can be successfully synthesized by a facile electrospinning method followed by pyrolysis treatment at 700°C. This material as anode exhibits high specific reversible capacity of 614 mAh·g^−1^ (0.27 mAh·cm^−2^), excellent rate performance, and long-cycle stability at 200 mA·g^−1^, which can be ascribed to the long-range continuous conductive carbon nanofibers and TiO_2_ nanoparticles with excellent structural stability and larger lattice of 0.363 nm than the radius of Na^+^. After almost 400 cycles, the capacity retention keeps ~100%, which indicates the high reversible performance and excellent tolerance of volume change in the process of Na^+^ insertion/extraction.

## Data Availability

The data used to support the findings of this study are included within the article.
